# Children Exhibit a More Comparable Neuromuscular Fatigue Profile to Endurance Athletes Than Untrained Adults

**DOI:** 10.3389/fphys.2019.00119

**Published:** 2019-02-15

**Authors:** Bastien Bontemps, Enzo Piponnier, Emeric Chalchat, Anthony J. Blazevich, Valérie Julian, Olivia Bocock, Martine Duclos, Vincent Martin, Sébastien Ratel

**Affiliations:** ^1^Clermont-Auvergne University, AME2P, Clermont-Ferrand, France; ^2^Centre for Exercise and Sports Science Research, School of Medical and Health Sciences, Edith Cowan University, Joondalup, WA, Australia; ^3^Clermont-Ferrand University Hospital, Clermont-Ferrand, France

**Keywords:** central fatigue, peripheral fatigue, growth, young people, high-intensity exercise

## Abstract

The present study compared neuromuscular fatigue profiles between children, untrained adults and adult endurance athletes during repeated maximal muscle contractions. Eighteen prepubertal boys, 19 untrained men and 13 endurance male athletes performed 5-s maximal voluntary isometric knee extensor contractions (MVICs) interspersed with 5-s recovery until MVIC reached 60% of its initial value. Single and doublet magnetic stimulations were delivered to the femoral nerve to quantify the time course of potentiated twitch amplitude (T_tw,pot_), high-frequency torque (T_100__Hz_) and the low-to-high frequency torque ratio (T_10__Hz_/T_100__Hz_), i.e., indicators of peripheral fatigue. M-wave-normalized EMG amplitudes (EMG/M) and the maximal voluntary activation level (VA) were calculated to quantify central fatigue. Adults (15.9 ± 3.9 repetitions) performed fewer MVICs than children (40.4 ± 19.7) and endurance athletes (51.7 ± 19.6), however, no difference was observed between children and athletes (*P* = 0.13). T_tw,pot_ (∼52%, *P* < 0.001), T_100__Hz_ (∼39%, *P* < 0.001) and T_10__Hz_/T_100__Hz_ (∼23%, *P* < 0.001) decreased only in adults. Similar decrements in vastus medialis and vastus lateralis EMG/M were observed in children and endurance athletes (range: 40–50%), and these were greater than in adults (∼15%). Whilst VA decreased more in children (-38.4 ± 22.5%, *P* < 0.001) than endurance athletes (-20.3 ± 10.1%, *P* < 0.001), it did not change in adults. Thus, children fatigued more slowly than adults and as much as endurance athletes. They developed less peripheral and more central fatigue than adults and, although central fatigue appeared somewhat higher in children than endurance athletes, both children and endurance athletes experienced greater decrements than adults. Therefore, children exhibit a more comparable neuromuscular fatigue profile to endurance athletes than adults.

## Introduction

It has been recently shown that prepubertal children fatigue much less than untrained young adults but as much as well-trained adult endurance athletes in a brief (30-s) bout of maximal cycling exercise (Wingate test) (Birat et al., 2018). In line with this, the amount of energy derived from oxidative metabolism during the 30-s all-out cycle sprint was found to be similar in children and endurance athletes, and (∼10%) more than in untrained adults. Additionally, the rate at which oxygen uptake declined after the exercise was similar in children and endurance athletes, and the rates at which heart rate returned to normal and lactate cleared from the blood were even faster in children. This suggests a similar, rapid recovery profile in both children and endurance athletes. Thus, the fatigue rates in response to whole-body high-intensity exercise appear to be comparable in children and endurance athletes, and this was associated with an incredible generation of energy derived from aerobic energy sources.

However, while some aspects of fatigue were compared between children, endurance athletes and untrained adults, neuromuscular function was not examined. Such a comparative analysis between groups may be requisite to further investigate the development and etiology of neuromuscular fatigue with respect to muscle phenotype (Colliander et al., 1988; Hamada et al., 2000). It was suggested that prepubertal children and endurance athletes experience similar peripheral (i.e., muscular) fatigue during high-intensity exercise but lower peripheral fatigue than untrained adults (Ratel and Blazevich, 2017). This is consistent with reports of prepubertal children fatiguing less at the peripheral level than untrained adults at least during sustained or repeated maximal voluntary isometric contractions (MVICs) (Streckis et al., 2007; Ratel et al., 2015). This has also been observed in endurance-trained athletes when compared to explosive power-trained athletes (Garrandes et al., 2007) or untrained adults (Bachasson et al., 2016). Experimental data derived from *in vivo* and *in vitro* muscle measurements in prepubertal children, untrained adults and endurance athletes appear to support this assumption. Prepubertal children are characterized by markedly higher muscle oxidative potential than young adults (Ratel et al., 2008; Tonson et al., 2010), and the same has been shown to be true for well-trained adult endurance athletes compared with untrained adults (Hug et al., 2005). Furthermore, comparisons of prepubertal children and well-trained adult endurance athletes indicate that they may have the same muscle phenotype (Ratel and Blazevich, 2017), i.e., type I muscle fiber percentage (Tesch and Karlsson, 1985; Lexell et al., 1992), mitochondrial volume density (Hoppeler et al., 1973; Bell et al., 1980) and succinate dehydrogenase activity (Gollnick et al., 1972; Eriksson and Saltin, 1974). However, scientific evidence for the relative rates of peripheral fatigue development during repeated maximal muscle contractions in prepubertal children and endurance athletes remains to be presented.

While clear evidence is not yet available, some data also indicate that prepubertal children may develop greater central fatigue (i.e., neural fatigue) during muscle contractions than untrained adults (Streckis et al., 2007; Ratel et al., 2015; Piponnier et al., 2018). This may be attributed to their ability to maintain exercise for longer, which then taxes the central nervous system. During repeated knee extensor (KE) MVICs performed to the same level of exhaustion the number of repetitions was found to be higher in prepubertal children (Armatas et al., 2010; Ratel et al., 2015), which may promote the development of central fatigue (Behm and St-Pierre, 1997). This could be also true in endurance adult athletes as they fatigue more slowly during submaximal contractions than untrained (Bachasson et al., 2016) or power-trained adults (Pääsuke et al., 1999). Furthermore, this greater central fatigue in children than untrained adults could represent a safety mechanism to prevent any extensive peripheral fatigue as has been previously proposed in ultra-endurance athletes. Taken together, the current evidence suggests that both prepubertal children and endurance athletes could develop less peripheral and more central fatigue during repeated MVICs than untrained adults and would hence have the same neuromuscular fatigue profile. However, direct scientific evidence showing such a result is still lacking.

Thus, the aim of the present study was to determine whether prepubertal children exhibit a comparable neuromuscular fatigue profile to adult endurance athletes during repeated maximal voluntary isometric contractions. We hypothesized that prepubertal children would fatigue less than untrained adults but similarly to endurance athletes. It is also presumed that the etiology of neuromuscular fatigue is comparable between children and endurance athletes, but they experience less peripheral and more central fatigue than untrained adults.

## Materials and Methods

### Ethics Statement

This study was approved by a local Institutional Ethics Review Board (Protection Committee of People for Biomedical Research South-East VI; authorization number, AU 1268) and conformed to the standards of use of human participants in research as outlined in the sixth Declaration of Helsinki, except for registration in a database. All the participants were informed of the experimental procedures and gave their written consent before any testing was conducted. In addition, the written consent of the parents/guardians was obtained for the children.

### Participants

Eighteen healthy prepubertal boys (8–11 years), 19 untrained men (18–30 years) and 13 highly trained endurance male athletes (21–45 years) volunteered to participate in the study. To be included, boys and untrained men had to perform recreational physical activity for ≤ 4 h per week and to be free of any medical contra-indication to physical activity. None of them were involved in any vigorous physical activity or engaged in a specific aerobic training program. Boys were recruited from primary and secondary schools while untrained men were university students. Their recreational activities were Alpine skiing, snowboarding, skateboarding, sailing, climbing, etc. In contrast, endurance-trained athletes were engaged in long distance physical activities for ≥ 6 times a week for at least 2 years and were international-class competitive athletes (i.e., marathon, triathlon, ultra-trail). All athletes could complete a 10,000 m race in 28–33 min. They were recruited from local athletics clubs.

### Experimental Procedures (Design)

All participants were tested in two experimental sessions separated by at least 48 h. During the first experimental session, anthropometric characteristics and maturation status were evaluated. Furthermore, a medical practitioner (pediatrician for the children) performed a clinical examination before familiarization with the subsequent experimental procedures. At the end of this session, participants were asked to perform several KE MVICs at knee angles of 30, 50, 60, 70, 75, 80, 85, 90, and 100° (0° = full extension) in a randomized order in order to determine the optimal angle for maximal KE torque production.

During the second session, all participants performed an intermittent voluntary exercise protocol with the KE muscles (see below for details). Before any exercise, volunteers performed a progressive warm-up (4 contractions up to ∼50% MVIC, 4 contractions up to ∼80% MVIC, and 2 contractions up to ∼100% MVIC). In addition, prior to the exercise protocol, participants performed three KE MVICs and two knee flexor MVICs with 2-min of rest between each MVIC. In order to prevent any extensive fatigue, an additional 5-min rest was allowed prior to the exercise protocol.

### Anthropometric Measurements and Maturation Assessment

A digital weight scale (TANITA, BC-545N, Japan) was used to measure body mass to the nearest 0.1 kg and barefoot standing height was assessed to the nearest 0.1 cm with a wall-mounted stadiometer (TANITA, HR001, Japan). Body mass index (BMI) was calculated as body mass (kg) divided by height squared (m^2^).

Two methods were used to assess children’s maturation: (1) Tanner stages were determined from a self-reported assessment on the basis of pubic hair and testicular/penis development, the children being assisted by their parents while completing the questionnaire; (2) Age from peak height velocity was used to assess somatic maturity and determined using standing height, sitting height, and body mass. Its calculation was based on sex-specific regression equations according to the method proposed by Mirwald et al. (2002).

### Intermittent Voluntary Exercise Protocol

Participants performed an intermittent voluntary exercise protocol consisting of 5-s KE MVICs interspersed with 5-s passive recovery periods until the voluntary torque reached the target value of 60% of its initial value for three consecutive MVICs. Participants were not informed of this criterion of task failure and had no feedback of torque output during the exercise in order to minimize pacing or targeting strategies. Participants were strongly encouraged by the investigators during each maximal effort throughout the experimental protocol. The number of repetitions was considered as the main indicator quantifying global fatigue. In order to evaluate the peripheral (i.e., muscular) and central (i.e., neural) components of fatigue, evoked mechanical and electromyographic (EMG) responses were measured throughout the exercise (see below for details).

### Torque Measurements

Isometric voluntary and evoked contractions were assessed using an isokinetic dynamometer (Biodex System 3, Biodex, Shirley, NY, United States). Participants were comfortably positioned on an adjustable chair with the hip joint flexed at 60° (0° = neutral position). The knee joint was fixed at the optimal angle for maximal KE torque production, which was determined during the first session (78.1 ± 5.2°, 74.5 ± 5.0°, and 75.8 ± 6.1° in children, untrained adults and endurance athletes, respectively). No significant difference was observed between groups. The rotation axis of the dynamometer was aligned with the lateral femoral condyle of the right femur. Moreover, the dynamometer lever arm was attached 1–2 cm above the lateral malleolus with a Velcro strap, to reduce cushioning and improve torque transmission and resolution. During each MVIC, participants were instructed to grip the lateral handle of the seat to stabilize the pelvis. Torque data were corrected for gravity, digitized and exported at analog-to-digital converter rate of 2 kHz PowerLab 8/35; ADInstruments, Bella Vista, NSW, Australia) driven by the LabChart 7.3 Pro software (ADInstrument, Bella Vista, NSW, Australia).

### EMG Recordings

EMG signals from vastus lateralis (VL), vastus medialis (VM), rectus femoris (RF), and biceps femoris (BF) were recorded during voluntary and evoked contractions using surface bipolar electrodes (Ag-AgCl, Blue Sensor N-00-S, Ambu, Denmark). The recording electrodes were taped lengthwise on the skin over the muscle belly with an inter-electrode distance of 20 mm according to the SENIAM recommendations. Low impedance (Z < 5 kΩ) at the skin-electrode surface was obtained by shaving, gently abrading the skin with a sandpaper and cleaning with alcohol. EMG signals were amplified (Dual BioAmp, ML 135, ADInstruments, Bella Vista, NSW, Australia) within a bandwidth frequency ranging from 10 to 500 Hz (common mode rejection ratio > 85 dB, gain = 1000), and simultaneously digitized together with torque signals (sampling rate: 2000 Hz).

### Femoral Nerve Stimulation

Knee extensor muscles were stimulated using magnetic stimuli delivered to the femoral nerve using a 70-mm figure-of-eight coil connected to two magnetic stimulators (Magstim 200^2^, Magstim, Whiteland, Dyfed, United Kingdom), both linked by the Bistim^2^ module (peak magnetic field strength 2.5 T, stimulus duration 115 μs; Magstim, Whiteland, Dyfed, United Kingdom). The coil was placed high in the femoral triangle in relation to the femoral nerve. Small spatial adjustments were initially performed to determine the optimal position where the greatest unpotentiated KE twitch amplitude (T_tw,unpot_) and the greatest compound KE muscle action potentials (i.e., maximal M-wave, M_max_) were evoked. Recruitment curves were used, prior to testing procedure, to determine the optimal stimulation intensity (i.e., the intensity where maximal T_tw,unpot_ and concomitant VL, VM, and RF M-waves amplitudes started to plateau). Briefly, two single stimulations were delivered every 20 s at 70, 80, 85, 90, 95, 97, and 100% of the maximal stimulator power output. T_tw,unpot_ and M_max_ plateaued at 92.6 ± 7.1%, 88.4 ± 11.4%, and 88.4% ± 12.3% in children, untrained adults, and endurance athletes, respectively. During subsequent testing, the stimulation intensity was set to 100% of the maximal stimulator power output to overcome the potential cofounding effect of axonal hyperpolarization (Burke, 2002). The intensity corresponded to 107.2 ± 8.4%, 113.0 ± 17.6%, and 115.4 ± 17.9% of the optimal intensity in children, untrained adults and endurance athletes, respectively. These supra-maximal intensities were statistically higher than the optimal stimulation intensities (*P* < 0.001) but were not statistically different between groups (*p* = 0.29).

### Peripheral Fatigue Indicators

In order to examine the time course of peripheral fatigue, amplitudes of potentiated single twitches (T_tw,pot_) were measured before, every five MVIC, and after the last MVIC of the exercise protocol. Twitches were systematically evoked 3 s after the cessation of MVIC. This allowed to obtain a potentiated mechanical response and hence reduce the variability in the calculation of maximal voluntary activation level (see below) (Kufel et al., 2002). Concomitant peak-to-peak M-wave amplitudes (M) were measured from VL, VM, and RF. Doublets at 10 and 100 Hz (T_10__Hz_ and T_100__Hz_) were also evoked before and immediately after the exercise protocol. Intensity of double stimulations was set to 60% of the maximal stimulator power output. Furthermore, the T_10__Hz_/T_100__Hz_ ratio was calculated to investigate low frequency fatigue [i.e., an estimate of the excitation-contraction (E-C) coupling failure].

### Central Fatigue Indicators

VL, VM, and RF EMG data were smoothed using a symmetric moving root-mean-square filter with a 500-ms window, and the peak torque prior to superimposed stimulation was recorded. This EMG value was normalized to the corresponding peak-to-peak amplitude of the M-wave (EMG/M) to account for differences in muscle mass and potential changes/differences in sarcolemmal excitability (Ratel et al., 2015).

The twitch interpolation technique was also used to estimate the maximal voluntary activation level (VA). Superimposed single twitch (T_tw,s_) and T_tw,pot_ were evoked during MVIC after the torque had reached a plateau and 3 s after MVIC cessation, respectively. This procedure was completed before, every fifth MVIC and after the last MVIC of the exercise protocol. Superimposed and potentiated torques allowed the quantification of VA, according to Merton’s equation (Merton, 1954):

$$\text{VA}\left(\%\right)=\left[1-\left({\text{T}}_{\text{tw},\text{s}}\cdot {\text{T}}_{\text{tw},\text{pot}}^{-1}\right)\right]\cdot 100$$

Finally, the level of antagonist activity (EMG_anta_) was determined every fifth MVIC using the following equation:

$${\text{EMG}}_{\text{anta}\;}\left(\%\right)=\left({\text{RMS}}_{\text{con}}\cdot {\text{RMS}}_{\text{max}}^{-1}\right)\cdot 100$$

where RMS_con_ is the RMS value of the BF muscle during intermittent contractions, and RMS_max_ is the maximal RMS value of the BF muscle during maximal voluntary knee flexion, recorded before the exercise protocol.

### Statistical Analysis

In order to compare changes across the exercise protocol between groups, variables measured during the exercise protocol were linearly interpolated between the nearest values at 20, 40, 60, and 80% of total number of repetitions (%REP). Values at the start (0%REP) and end (100%REP) of exercise were taken as pre- and post-fatigue values, respectively.

Data were screened for normality of distribution and homogeneity using a Shapiro–Wilk test and the Bartlett test, respectively. Age, anthropometric characteristics and total number of repetitions were compared between groups using a one-way ANOVA. Differences in absolute values and changes relative to the initial values for voluntary and evoked torque, EMG and VA variables were analyzed using a two-way (group × %REP) ANOVA with repeated measures. When ANOVA revealed significant main or interaction effects, a Tukey HSD *post hoc* test was applied to test the discrimination between means. The effect size and statistical power have also been reported when significant main or interaction effects were detected. The effect size was assessed using the partial eta-squared (η^2^) and ranked as follows: ∼ 0.01 = small effect, ∼ 0.06 = moderate effect, ≥0.14 = large effect (Cohen, 1969). Linear regression models were used to determine correlations between T_tw,pot_, EMG_anta_ and VA changes over the course of the fatigue protocol. Statistical significance was set at *P* < 0.05. Statistical procedures were performed using Statistica 8.0 software (StatSoft, Inc., United States). Results are presented as mean ± standard deviation (SD) in text and table.

## Results

### Participants’ Physical Characteristics

The participants’ physical characteristics are described in Table 1. As expected, children showed significantly lower values for age, height, body mass and BMI compared to adults and endurance athletes (*P* < 0.001). Furthermore, compared to untrained adults, endurance athletes were older but displayed the same height, body mass and BMI. All the children were prepubertal (Tanner stages I and II). The maturity onset was -3.4 ± 0.6 years and their age at the peak height velocity was 13.5 ± 0.4 years.

**Table 1:** Physical characteristics of children (C), adults (A) and endurance athletes (EA).

	C	A	EA
	(*n* = 18)	(*n* = 19)	(*n* = 13)
Age (y)	10.4 ± 0.8	21.7 ± 3.4***	32.7 ± 8.1***;^§§§^
Body mass (kg)	33.0 ± 5.2	71.9 ± 8.7***	70.5 ± 5.4***
Height (cm)	138.9 ± 6.6	178.2 ± 7.6***	179.4 ± 5.8***
BMI (kg/m^2^)	17.0 ± 1.4	22.6 ± 2.2***	21.9 ± 1.5***


*^∗∗∗^Significantly different from children at p < 0.001; ^§§§^ significantly different from adults at p < 0.001. BMI, body mass index.*

### Global Fatigue

#### Number of Repetitions

ANOVA revealed a significant group effect regarding the number of repetitions [*F*(2;47) = 22.56, *P* < 0.001, η^2^ = 0.87, power = 0.99], which were significantly lower in untrained adults (15.9 ± 3.9 repetitions) than children (40.4 ± 19.7, *P* < 0.001) and endurance athletes (51.7 ± 19.6, *P* < 0.001). However, no significant difference was found between children and endurance athletes (*P* = 0.13).

#### MVIC Torque

**FIGURE 1:**
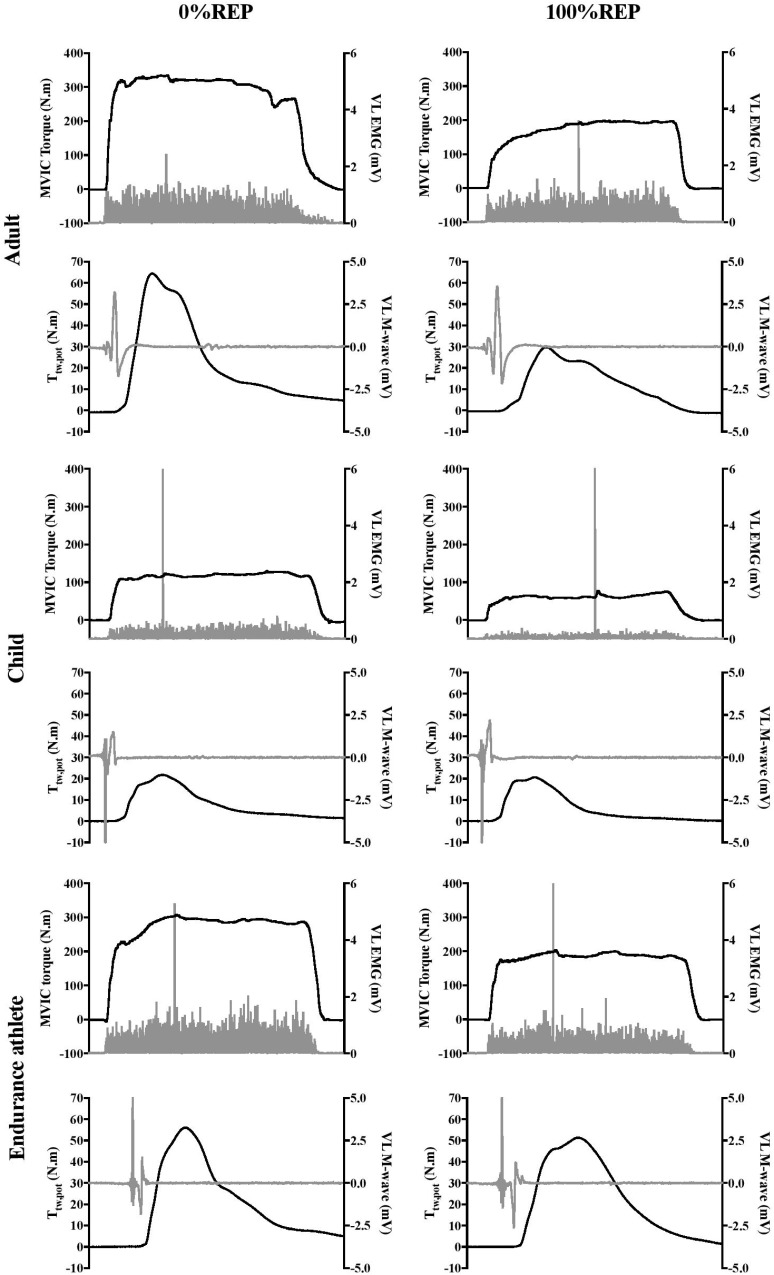
Example of raw data for MVIC torque, T_tw,pot_, VL EMG, VL M-wave obtained at 0%REP and 100%REP in a typical adult **(top)**, child **(intermediate),** and endurance athlete **(bottom)**. Greater reduction in twitch torque (T_tw,pot_) in adults and in EMG in children and endurance athletes can be observed.

A significant interaction effect (group × %REP) was found for absolute MVIC torque [*F*(10;235) = 48.68, *P* < 0.001, η^2^ = 0.67, power = 1.0]. As expected, adults and endurance athletes produced significantly higher absolute MVIC torque values than children (*P* < 0.001). Initial values were 100.8 ± 20.8, 319.9 ± 48.6, and 297.4 ± 38.3 N.m in children, adults and endurance athletes, respectively. Furthermore, ANOVA showed a significant interaction effect (group × %REP) for MVIC torque when expressed as percentage of initial value [*F*(8;188) = 5.10, *P* < 0.001, η^2^ = 0.18, power = 0.99]. MVIC torque significantly and progressively decreased over the exercise protocol in the three groups. However, while children and endurance athletes showed the same time-course in MVIC torque decrement, children displayed a greater decrease at 20%REP than untrained adults (*P* < 0.001). An example of raw data for the MVIC torque obtained at 0%REP and 100%REP in a typical adult (top panel), child (intermediate panel) and endurance athlete (bottom panel) is presented in Figure 1.

### Peripheral Fatigue

#### Twitch Torque

**FIGURE 2:**
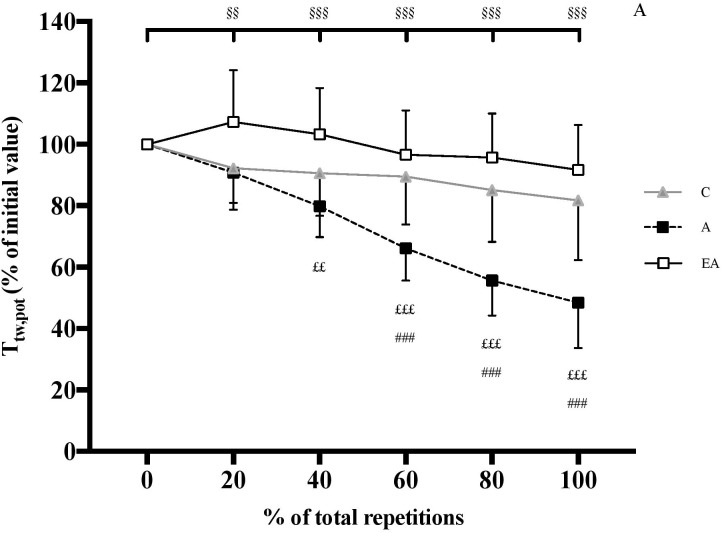
Time course of potentiated twitch torque (T_tw,pot_) of the knee extensor (KE) muscles (expressed as percentage of the initial value) during the exercise protocol (expressed as percentage of total repetitions) in children (C), adults (A) and endurance athletes (EA). §§ and §§§: significantly different from the first MVC at *P* < 0.01 and *P* < 0.001, respectively; ###: significantly different between C and A at *P* < 0.001; ££ and £££: significantly different between EA and A at *P* < 0.01 and *P* < 0.001, respectively.

ANOVA revealed a significant interaction effect (group × %REP) for absolute T_tw,pot_ [*F*(10;235) = 50.63, *P* < 0.001, η^2^ = 0.68, power = 1.0]. Adults and endurance athletes showed significantly higher absolute T_tw,pot_ than children over the exercise protocol (*P* < 0.001). Initial values were 19.8 ± 4.2, 66.5 ± 11.0 and 51.4 ± 12.2 N.m in children, adults and endurance athletes, respectively. Whilst T_tw,pot_ significantly decreased in adults (-51.6 ± 14.8%, *P* < 0.001), it remained unchanged in children and endurance athletes (Figure 2).

#### Doublet Torque

**FIGURE 3:**
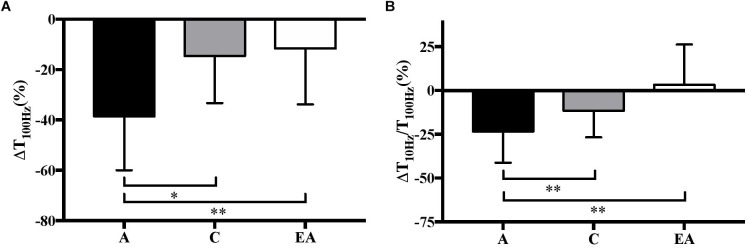
Relative variations (%) in high-frequency torque (ΔT_100Hz_) **(A)** and low- to high-frequency torque ratio (ΔT_10__Hz_/T_100__Hz_) **(B)** in children (C), adults (A) and endurance athletes (EA). ^∗∗^ and ^∗∗∗^: significantly different at *P* < 0.01 and *P* < 0.001, respectively.

ANOVA revealed a significant interaction effect (group × %REP) for absolute T_100Hz_ [*F*(2;40) = 11.74, *P* < 0.01, η^2^ = 0.37, power = 0.99] and T_10__Hz_/Tt_100__Hz_ [*F*(2;40) = 6.20, *P* < 0.01, η^2^ = 0.24, power = 0.87]. T_100__Hz_ and T_10__Hz_/T_100__Hz_ only decreased in adults (-38.6 ± 21.4 and -23.4 ± 17.8%, *P* < 0.001, respectively; Figure 3).

#### M Wave Amplitudes

VL, VM and RF M-wave remained unchanged throughout the exercise protocol in children, adults, and endurance athletes.

### Central Fatigue

#### Normalized EMG Amplitudes

**FIGURE 4:**
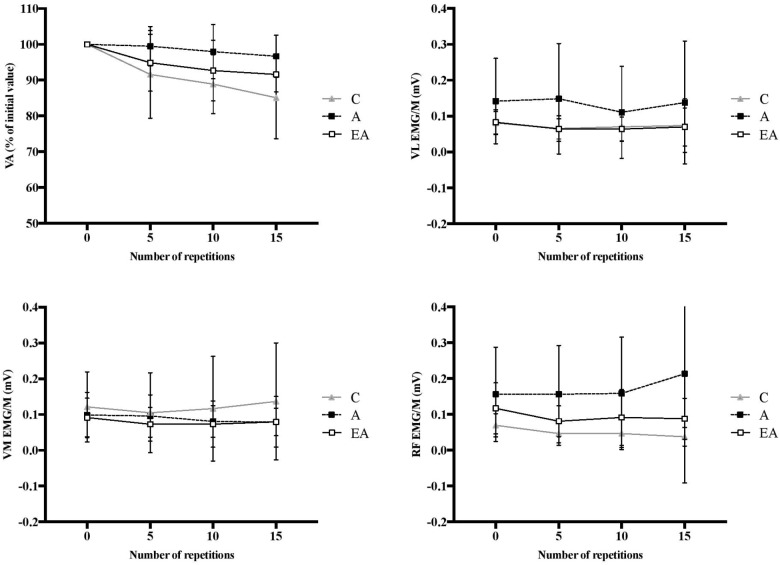
Time course of VA, EMG/M of VL, VM and RF muscles over the first 15 muscle contractions (i.e., at or near the point of failure in adults) in children (C), adults (A) and endurance athletes (EA). No significant interaction effects (group × contractions) were observed.

A significant group effect was found for the relative changes in EMG/M for VM [*F*(2;52) = 5.34, *P* < 0.01, η^2^ = 0.19, power = 0.81] and RF [*F*(2;52) = 9.51, *P* < 0.001, η^2^ = 0.30, power = 0.97]. In contrast, no significant group effect was found for VL EMG/M decreased to a greater extent in children than adults in VM (-46.2 ± 24.1 vs. -14.0 ± 28.0%, respectively, *P* < 0.05) and RF (-55.2 ± 26.5 vs. -15.6 ± 20.8%, respectively, *P* < 0.001). No difference in the VM EMG/M changes was found between children and endurance athletes, although a tendency was detected in RF EMG/M toward greater reduction in children (-55.2 ± 26.5 vs. -39.7 ± 14.9%, respectively, *P* = 0.075). No significant interaction effect (group × contractions) was observed for VM, VL, and RF EMG/M after 15 MVICs, i.e., at (or near) the point of failure in adults (Figure 4).

#### Voluntary Activation

**FIGURE 5:**
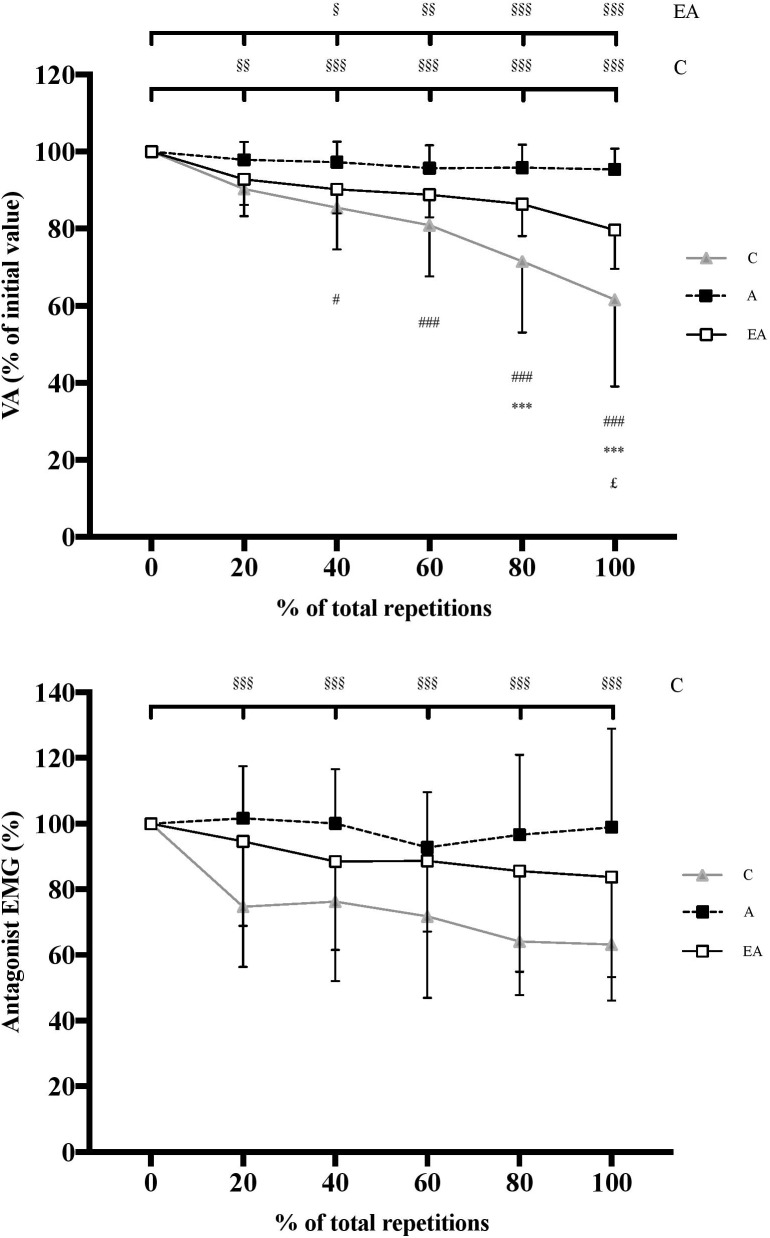
Time course of voluntary activation level (VA) of the knee extensors **(top)** and antagonist EMG of the biceps femoris **(bottom)** (expressed as percentage of the initial value) during the exercise protocol (expressed in percentage of total repetitions) in children (C), adults (A) and endurance athletes (EA). §, §§, and §§§: significantly different from the first MVIC at *P* < 0.05, *P* < 0.01, and *P* < 0.001, respectively; ^∗∗∗^: significantly different between C and EA at *P* < 0.001; # and ###: significantly different between C and A at *P* < 0.05 and *P* < 0.001, respectively; £: significantly different between EA and A at P < 0.05.

Significant interaction effect (group × %REP) was observed for absolute VA [*F*(10;235) = 12.60, *P* < 0.001, η^2^ = 0.35, power = 1.0]. No significant difference was observed between groups at 0%REP, with absolute initial VA of 90.1 ± 6.3%, 92.8 ± 3.9%, and 94.5 ± 2.9% in children, adults and endurance athletes, respectively. No significant change in VA was observed in untrained adults throughout the exercise protocol. However, children and endurance athletes displayed a significant and progressive decrement until the end of the test (*P* < 0.001) (Figure 5), although the decrement was greater in children (-38.4 ± 22.5%) than endurance athletes (-20.3 ± 10.1%, *P* < 0.001). No significant correlation was found between the changes in T_tw,pot_ and VA over the fatigue test whatever the population studied.

No significant interaction effect (group × contractions) was observed for VA after 15 MVICs, i.e., at (or near) the point of failure in adults (Figure 4).

#### Antagonist EMG Amplitudes

A significant interaction effect (group × %REP) was observed for EMG_anta_ [*F*(10;205) = 4.99, *P* < 0.001, η^2^ = 0.20, power = 0.99]. Children showed significantly higher initial EMG_anta_ than adults (17.3 ± 6.0 vs. 7.2 ± 4.6%, respectively, *P* < 0.01). In contrast, no significant difference in initial EMG_anta_ was found between children and endurance athletes (11.6 ± 8.7%). Whilst no significant change in EMG_anta_ was observed in adults and endurance athletes throughout the exercise protocol, children displayed a significant decrement (-36.8 ± 17.0%; *P* < 0.001) (Figure 5). No significant correlation was found between the changes in EMG_anta_ and VA over the course of the fatigue test whatever the population studied.

## Discussion

The aim of the present study was to determine whether prepubertal children exhibit a comparable neuromuscular fatigue profile to highly trained adult endurance athletes during repeated maximal voluntary isometric contractions. The results largely confirm our hypothesis. Prepubertal children fatigued more slowly than untrained young adults and as much as highly trained adult endurance athletes. They also developed less peripheral and more central fatigue than untrained adults and about the same as the endurance athletes, although some evidence indicated a slightly greater central fatigue in children.

### Comparison Between Children and Untrained Adults

The results confirm that children fatigue more slowly than adults over repeated MVICs. This finding is consistent with the available evidence showing a higher number of repetitions to task failure (i.e., a predetermined decrement in force) in prepubertal children than adults (Armatas et al., 2010; Ratel et al., 2015), and cannot be explained by differences in physical activity patterns as the same inclusion criteria were applied to both groups. Regarding peripheral (muscular) function, the greater decrement in T_tw,pot_, T_100__Hz_ and T_10__Hz_/T_100__Hz_ in adults (Figure 2, 3) suggests that they developed more peripheral fatigue than prepubertal children, as previously reported (Streckis et al., 2007; Hatzikotoulas et al., 2014; Murphy et al., 2014; Ratel et al., 2015; Piponnier et al., 2018). Moreover, the absence of change in T_tw.pot_ and T_10__Hz_/T_100__Hz_ in prepubertal children suggests that E-C coupling was not affected. The preservation of M-wave characteristics in children also suggests that action potential transmission was maintained, although an alteration occurring within the T-tubules cannot be excluded. Taken together, the data indicate that the peripheral mechanisms of force production were less affected by the fatiguing exercise in children than untrained adults.

Nonetheless, prepubertal children showed significant decrements in M-wave normalized EMG (in VM and RF) and VA over the course of the fatiguing exercise (Figure 4), while only a small (and statistically lesser) decrease was observed in adults. These changes indicate a significant central fatigue response in children. This might account for their lesser fatigue at the peripheral level because the central nervous system may limit the recruitment of motor units to prevent extensive homeostatic disturbance, muscle damage, or other biological harm (Noakes et al., 2005). However, we need to be cautious with this hypothesis since no significant relationship between the changes in twitch torque and voluntary activation level was observed over the course of the fatiguing exercise in children. It is also possible that the progressive decrease in the activation level of the biceps femoris in children may have contributed to limit the loss of KE torque, and therefore delay the performance decline. However, this assertion remains to be confirmed since no significant correlation was found between agonist and antagonist muscle activities throughout the fatigue test. An alternative hypothesis is that the longer exercise duration in prepubertal children triggered the development of central fatigue, as previously proposed by Behm and St-Pierre (1997) in their exploration of submaximal intermittent exercise. This possibility is supported in the current study by the fact that no significant differences in VA decrement or EMG/M of VL, VM or RF muscles were observed between children and adults after 15 MVICs, i.e., at the end of the exercise protocol in adults. After performing the same number of contractions, the magnitude of central fatigue was similar between groups; however, because children were able to perform more contractions than adults at task failure (60% of initial MVIC), they incurred a greater deficit in central (neural) function.

### Comparison Between Children and Highly Trained Adult Endurance Athletes

Contrary to untrained adults, endurance athletes performed approximately the same number of repetitions to task failure as prepubertal children. Athletes were highly trained in endurance since they could complete a 10,000 m race in 28–33 min. This finding is consistent with the data of Birat et al. (2018) showing that prepubertal children have a more comparable fatigue profile during a 30-s all-out cycle test to well-trained adult endurance athletes than untrained adults. This assertion is also supported by a similar peripheral fatigue in these groups. In fact, no significant change in T_tw,pot_, T_100__Hz_, T_10__Hz_/T_100__Hz_ or M-wave were found in the prepubertal children or highly trained adult endurance athletes (Figure 2, 3). The results might be attributed to a similar, more oxidative muscle phenotype in both populations (Ratel and Blazevich, 2017), i.e., type I muscle fiber percentage (Tesch and Karlsson, 1985; Lexell et al., 1992), mitochondrial volume density (Hoppeler et al., 1973; Bell et al., 1980) and succinate dehydrogenase activity (Gollnick et al., 1972; Eriksson and Saltin, 1974). This could attenuate the accumulation of hydrogen ions (H^+^) and inorganic phosphate (Pi) in exercising muscle, and therefore their deleterious effects on muscle force production (Nelson et al., 2014). Furthermore, the greater muscle oxidative capacity in children and endurance athletes compared to untrained adults could increase post-exercise phosphocreatine resynthesis and proton efflux rate, and thus the recovery of force output within each of the 5-s rest intervals between contractions throughout the repeated MVICs (Bernús et al., 1993; Hug et al., 2005; Tonson et al., 2010). This more oxidative profile in children and endurance athletes is supported by the data of Birat et al. (2018) showing a greater relative contribution of energy derived from aerobic metabolism during a 30-s all-out cycle sprint as well as faster post-exercise recovery rates of oxygen uptake, heart rate and lactate from the blood in children and endurance athletes compared to untrained adults. Therefore, prepubertal children might develop as much peripheral fatigue as highly trained adult endurance athletes during repeated maximal voluntary contractions owing to their comparable metabolic profile.

Regarding neural factors, VL and VM EMG/M decreases were statistically consistent in prepubertal children and endurance athletes, although a tendency toward greater reduction in RF EMG/M was detected in children. Furthermore, VA was significantly decreased in both children and endurance athletes, and a greater reduction was observed in children (Figure 4). The physiological underpinnings of the greater central fatigue in endurance athletes remain to be clarified. However, as observed in children, it is likely that longer exercise duration in endurance athletes compared to untrained adults due to reduced peripheral fatigue taxed the central nervous system. One argument is that VA and EMG/M were very similar at (or near) the point of failure in adults, suggesting that the rate of accumulation of central fatigue was the same as in untrained adults, and that the greater central fatigue at task failure resulted directly from the greater accumulation of work in endurance athletes. Regarding the central regulation of the antagonist coactivation, the decrease in biceps femoris activity in children occurred simultaneously with the reduction in agonist KE activation, which is consistent with the common drive theory (Psek and Cafarelli, 1993). It may have served to maintain the net joint torque production in children, and thus preserve joint integrity, which was not the case after fatigue in endurance athletes or adults due to the imbalance between changes in agonist and antagonist muscle activities. However, we need to be cautious with this interpretation since no significant correlation was found between the changes in EMG_anta_ and VA over the course of the fatigue test.

Several considerations should be mentioned in this study. Firstly, T_100__Hz_ was evoked using a submaximal intensity to reduce discomfort. The use of a submaximal intensity may bias the evaluation of T_100Hz_ (Martin et al., 2004); however, this limitation should similarly affect children and adults. Secondly, the use of a doublet with the twitch interpolation technique was found to improve the VA assessment (Gandevia, 2001) through a higher signal-to-noise ratio (Harridge and White, 1993) and/or a lesser variability in measurements (Martin et al., 1996). In the current study, for ethical reasons, it was not possible to use double stimuli because it is too painful for children. For this reason we used a single stimulation delivered at supramaximal intensity, as has been previously done in this population (Hatzikotoulas et al., 2014; Ratel et al., 2015; Piponnier et al., 2018).

## Conclusion

Prepubertal children fatigued more slowly than untrained young adults and as much as highly trained adult endurance athletes. They also developed less peripheral and more central fatigue than untrained adults and about the same as the endurance athletes, although some evidence indicated a slightly greater central fatigue in children. Therefore, children exhibited a more comparable neuromuscular fatigue profile to highly trained adult endurance athletes than untrained adults.

## Translational Perspective

The present results show that children’s muscles fatigue more slowly than those of untrained adults but in a similar way to those of adult endurance athletes, which indicates that they have similar neuromuscular profiles. Such data provide strong hints as to how to optimize exercise and sporting performance in children, and how their training might need to differ from adults. Muscle aerobic capacity is significant in children, yet their anaerobic capacity is possibly lower than in untrained adults (Eriksson and Saltin, 1974; Tonson et al., 2010). A greater benefit might therefore be derived from using shorter, higher-intensity exercise bouts in training to stimulate anaerobic adaptations in younger athletes. Nonetheless, as enjoyment of exercise may influence motivation to exercise, from a public health perspective young non-athletes might be encouraged to perform sports or exercise activities in which repeated efforts predominate, which can be well performed by the children, in order to maintain physical activity (and health) levels. Adults (and adolescents), on the other hand, may need to emphasize physical training methods that target muscle aerobic capacity improvements and fatigue resistance. One might also speculate as to the possible health consequences of the loss in muscle aerobic capacity from childhood to adulthood (Taylor et al., 1997), and the capacity for aerobic exercise training to maintain it. Metabolic diseases, including diabetes, and many forms of cancer are increasing in prevalence in younger people but are still rarely seen in children (Gunn et al., 2016). Given that mitochondrial function is both an important factor influencing aerobic exercise capacity as well as metabolic disease and cancer risk (Fleischman et al., 2009; Kamp et al., 2011), it might be the case that the loss of muscle aerobic capacity between childhood and early adulthood is a key maturation step that allows metabolic diseases to take hold. Future study is required to more fully examine the link between maturation and disease, and to test whether the maintenance of “childhood muscles” through exercise training might be an effective disease prevention medicine.

## New and Noteworthy

• Prepubertal children develop less peripheral and more central fatigue than adults and, although central fatigue appears slightly higher in children than endurance athletes, both children and endurance athletes experience similar peripheral fatigue.
• Prepubertal children exhibit a more comparable neuromuscular fatigue profile to endurance athletes than adults.
• These data reveal why prepubertal children are able to produce repeated high-intensity exercise efforts with short rest periods when most of adults feel exhausted.

## Author Contributions

This study was conducted in the laboratory of metabolic adaptations during exercise in physiological and pathological conditions (AME2P, EA 3533) at the Clermont Auvergne University, France. BB, EP, VM, and SR designed the research. BB, EP, EC, VJ, OB, and MD collected the data and performed the research. BB, EP, EC, AB, VM, and SR analyzed the data. SR supervised the research. BB, EP, AB, and SR wrote the manuscript. All authors provided critical revisions important for intellectual content of the finished manuscript, approved the final version of the manuscript, and agree to be accountable for all aspects of the work in ensuring that questions related to the accuracy or integrity of any part of the work are appropriately investigated and resolved. All persons designated as authors qualify for authorship, and all those who qualify for authorship are listed.

## Conflict of Interest Statement

The authors declare that the research was conducted in the absence of any commercial or financial relationships that could be construed as a potential conflict of interest.
